# Contrast enhancement for early cancer imaging by Gd-nanoparticles and active feedback MRI

**DOI:** 10.1186/1470-7330-15-S1-P2

**Published:** 2015-10-02

**Authors:** S Ray, C-H Hsu, F-C Lin, Z Li, T Kim, Y-Y Lin

**Affiliations:** 1 Department of Chemistry of Biochemistry, University Of California, Los Angeles, 607 Charles E. Young Drive East, Los Angeles, CA 90095-1569, USA

## Aim

Early detection of high-grade malignancy using enhanced MRI techniques significantly increases not only the treatment options available, but also the patients’ survival rate. For this purpose, we have developed a new method, termed “Active-Feedback MRI”. An active feedback electronic device was homebuilt to implement active-feedback pulse sequences to generate avalanching spin amplification, which enhances the weak field originated from T_1_ contrast agents such as Gd-nanoparticles that target and label the cancer cells.

## Methods

The general principles of the “Active-Feedback MRI” can be found in our previous work (e.g., Science 290, 118, 2001). Here, its specific applications to image Gd-nanoparticles in early cancers were developed and demonstrated. (i) First, an active-feedback electronic device was home-built to generate feedback fields from the received FID current. The device is to filter, phase shift, and amplify the signal from the receiver coils and then retransmit the modified signal into the RF transmission coil, with adjustable and programmable feedback phases and gains. (ii) Next, an active-feedback pulse sequence was developed to enhance the contrast originated from local magnetic-field gradient variations due to Gd-nanoparticles.

## Results

*In vivo* subcutaneous glioblastoma multiforme (GBM) and cervical cancer mice models were imaged. While T_2_ parameter images, T_2_-weighted images, and T_1_-Gd-weighted images could not clearly locate the early cancers, our active-feedback images and decay constant mapping successfully highlight the early cancers with a close correlation with histopathology. Statistical results show that this new approach provides significant improvements in cancer detection sensitivity, as measured by “contrast-to-noise ratio” (CNR) or “Visibility”.

## Conclusion

*In vivo* subcutaneous xenografts GBM and cervical cancer mouse models validated the superior contrast/sensitivity and robustness of this approach towards early cancer detection. Spin dynamics and results from computer simulations will also be discussed.

